# The role of prognostic nutritional index for clinical outcomes of gastric cancer after total gastrectomy

**DOI:** 10.1038/s41598-020-74525-8

**Published:** 2020-10-15

**Authors:** Zhu Xishan, Zhao Ye, Ma Feiyan, Xuan Liang, Wu Shikai

**Affiliations:** 1 Oncology Department, Peking University, First Hospital, Cheniandianhutong No.5, Andingmen Street, DongCheng District, Beijing, China; 2 Pathology Department, Peking University, First Hospital, Beijing, China; 3 Radiotherapy Department, Chengde Medical College, Baoding No.1 Middle Hospital, Baoding, China

**Keywords:** Cancer, Cancer metabolism, Cancer prevention

## Abstract

The purpose of this article is to evaluate the relationship between the nutrition-based microenvironment and clinicopathological information for gastric cancer patients and to investigate the prognostic value of nutrition index for gastric cancer patients undergoing total gastrectomy. We retrospectively collected clinical information of 245 gastric cancer patients who underwent total gastrectomy in our hospital between January 1st 2005 and December 30th 2015. According to the prognostic nutritional index (PNI) level, they were divided into low PNI (< 43) group and high PNI (≥ 43) group. The relationship between PNI and the disease-free survival (DFS) and overall survival (OS) were analyzed by statistical analysis. Univariate analyses demonstrated that TNM stage (p = 0.025), patients age (p = 0.042), lymph node metastasis (p = 0.028), tumor differentiation (p = 0.037) and a low PNI (p = 0.033) were closely correlated with a poor prognosis. In multivariate analysis, TNM stage (p = 0.027) and a low PNI (p = 0.041) were found to be independently associated with poor survival. Additionally, when age was considered as a stratified factor, univariate analyses demonstrated that low PNI correlated with shorter DFS in non-elderly (< 65) patients (p = 0.022) and shorter DFS (p = 0.036) and OS (p = 0.047) in elderly (≥ 65) patients. The low prognostic nutritional index is an independent risk factor associated with poor gastric cancer survival which represents the nutritional microenvironment. Patients with low pre-operative prognostic nutritional index levels should be observed more closely after surgery to prevent the occurrence of post-operative complications in the near future.

## Introduction

Malignancy may be described as a state formed in the setting of specific tumor-host relationships at the molecular and cellular microenvironment levels^1^. The tumor microenvironment has many differences in physical and chemical properties from the normal internal environment of the human body. The most notable features are its low oxygen, low pH and high pressure. More and more researches indicate systemic nutrition has been found to be a crucial ingredient of the tumor microenvironment that plays remarkable roles in tumor growth, progression and metastasis^2^. For patients with malignant tumors, the tumor itself will consume a lot of protein, causing damage to tissue structure and organ functions; in addition, gastrointestinal obstruction caused by digestive organ tumors can lead to loss of appetite, nausea, vomiting, diarrhea which is also associated with poor nutrition and immunosuppression^3–5^.

The prognostic nutritional index (PNI) is calculated by the serum ALB (albumin) and the total number of peripheral blood lymphocytes and PNI has attracted more and more attention for its convenience and significance in clinical application. Albumin represents the nutritional condition of the human body and peripheral blood lymphocyte is an important immune index, the unbalance of albumin and lymphocyte is closely correlated with poor post-operative complications and cancer outcomes which have already demonstrated by multiple cancer types such as liver cancer^6^, non-small cell lung cancer^7^, bladder cancer^4^, pancreatic cancer^8,9^, colorectal cancer^10^, esophageal cancer^11–13^, ovarian cancer^14^, and renal cell carcinoma^15^. As far as we know, there is limited research on PNI in gastric cancer application.

Besides, gastric cancer is a group of heterogeneous tumors based on distinctive morphological and molecular genetic features which closely correlates with the nutritional conditions, peripheral blood cells might reflect the inflammatory and immune response of patients to malignant tumors and are critical for determining the treatment response and clinical outcomes of patients^16,17^.

As such, the present study aimed to evaluate the prognostic impact of PNI in patients with gastric cancer after radical gastrectomy. These results may reveal the important role of nutrition-based factors in gastric cancer after radical gastrectomy and may also help to evaluate patient prognosis.

## Results

### Example of histological and morphological characteristics in the gastrectomy tissues

**Figure 1:**
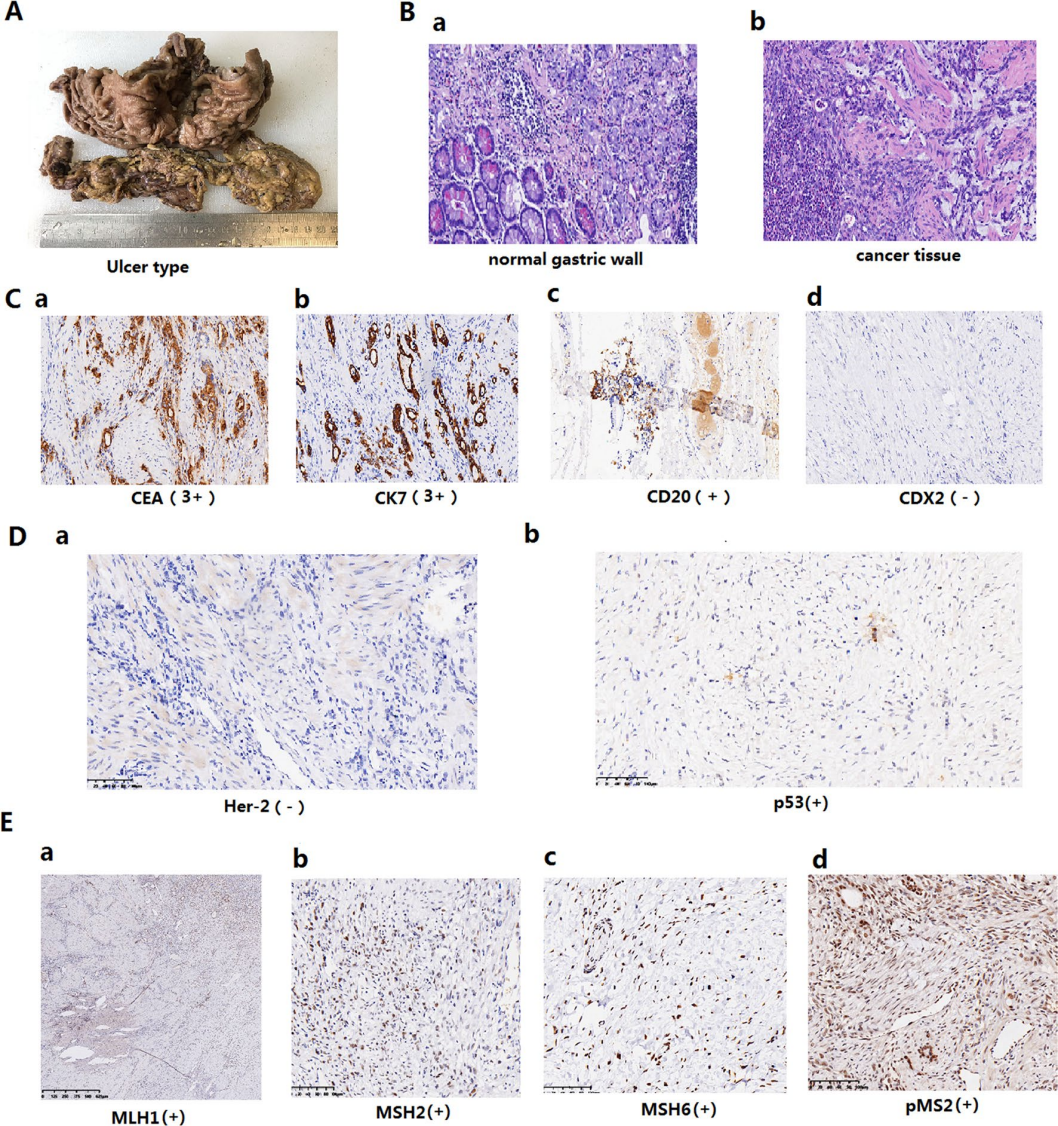
Histological and morphological characteristics in the gastrectomy tissues. (**A**) The general pathology of this case was ulcer type. (**B**) There was a clear contrast between the normal gastric wall and tumor tissue. (**C**) The tissue of this patient showed typical immunological markers of gastric cancer: CEA, CK7 and CD20 were all positive and CDX2 was negative. (**D**) The expression of Her-2 was negative and p53 was weakly positive. (**E**) The expression of MLH1, MSH2, MSH6 and pMS2 was all positive (p MMR).

We chose a gastric cancer patient randomly and the morphology, pathology and diagnostic markers were presented in Fig. 1. The general pathology of this case was ulcer type (Fig. 1A). There was a clear contrast between the normal gastric wall and tumor tissue (Fig. 1B). The tissue of this patient showed typical immunological markers of gastric cancer, that was, CEA, CK7 and CD20 were all positive, while CDX2 was negative (Fig. 1C). The other two very important markers for the pathological diagnosis of gastric cancer were Her-2 and p53. Gastric cancer patients with Her-2 positive should be treated with anti-Her-2 medicine, and p53 reflected tumor proliferation activity, that was, patients with high p53 had high tumor cell proliferation activity. In this case, the expression of Her-2 was negative (Fig. 1D) which suggested no need of anti-Her-2 treatment and p53 was weakly positive (Fig. 1D) which indicated normal tumor activity. Another important immunological marker of gastric cancer was the identification of mismatch repair which was shown by MLH1, MSH2, MSH6 and pMS2. The expression of MLH1, MSH2, MSH6 and pMS2 was all positive (pMMR) (Fig. 1E). So, immunotherapy was not the first choice for this patient. The results from Fig. 1 indicated that the proper and accurate pathological information was critical to clinical treatment decisions for gastric cancer.

### Correlations between the PNI and clinical characteristics

**Figure 2:**
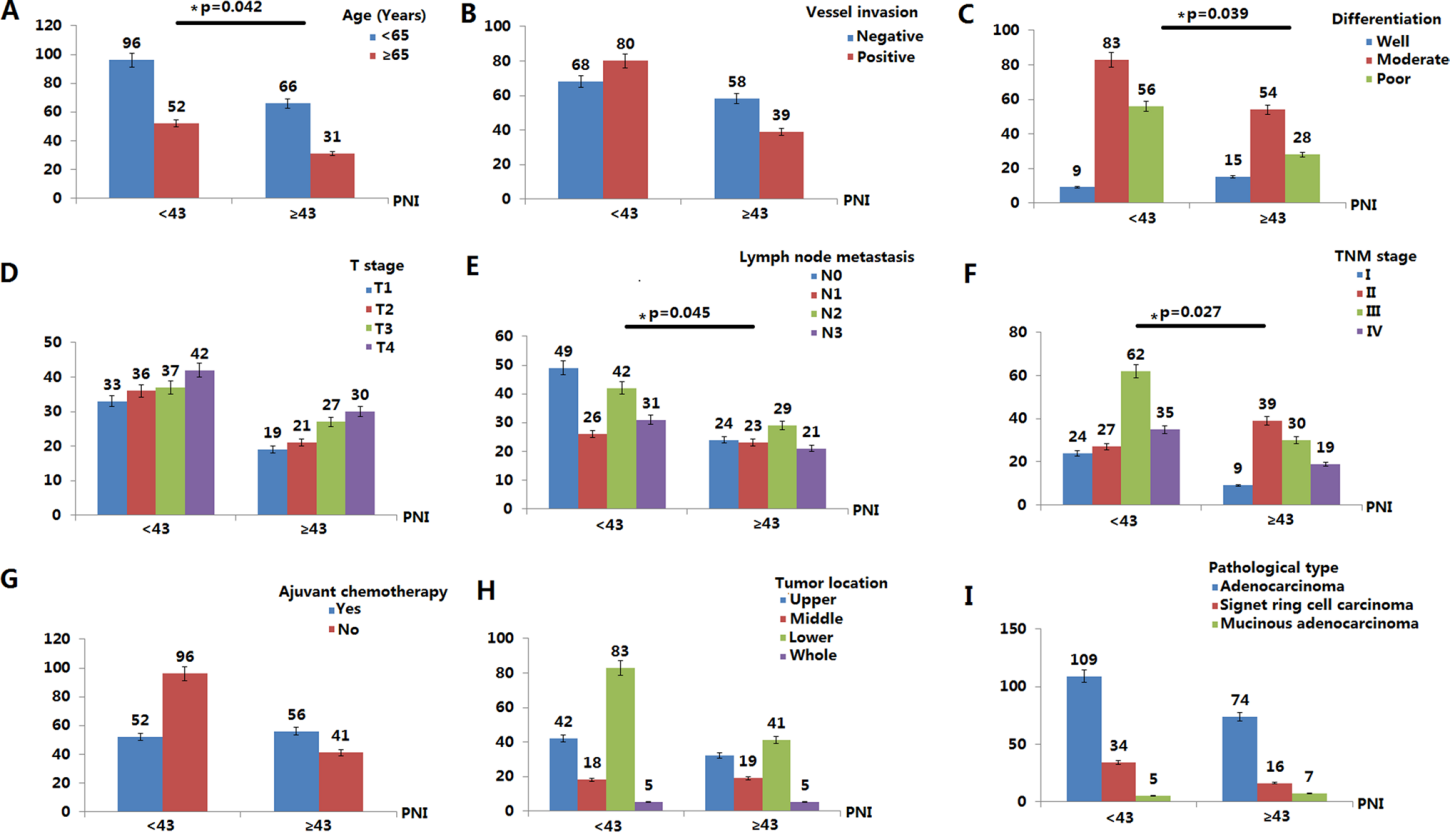
Relationship between PNI and clinicopathological features in 245 gastric cancer patients after total gastrectomy. Pre-operative PNI level was associated with TNM stage (p = 0.027), tumor differentiation (p = 0.039), patients age (p = 0.042) and lymph node metastasis (p = 0.045).

The patient characteristics are shown in Fig. 2. The PNI ranged from 33.9 to 52.4 (Supplementary Fig. 1), with a median level of 45.7 and the optimal cut-off point of the PNI was 43.15 in our research. So, the patients were divided into high PNI (PNI ≥ 43 n = 97, 39.6%) and low PNI groups (PNI < 43, n = 148, 60.4%). Correlations of clinical characteristics of the pre-operative PNI are summarized in Fig. 2. Pre-operative PNI level was associated with TNM stage (p = 0.027), tumor differentiation (p = 0.039), patients age (p = 0.042) and lymph node metastasis (p = 0.045).

### Predictive values of the PNI

Univariate statistical analyses demonstrated that TNM stage (hazard ratio [HR] 4.378; 95% confidence interval [CI] 2.581–6.175; p = 0.025), patients age (HR 2.116; 95% CI 0.493–4.739; p = 0.042), lymph node metastasis (HR 2.392; 95% CI 0.469–4.315; p = 0.028), tumor differentiation (HR 3.542; 95% CI 0.764–6.320; p = 0.037) and a low PNI (HR 2.573; 95% CI 0.692–4.454; p = 0.033) were significant risk factors for a poor prognosis (Table 1). In multivariate analysis, TNM stage (HR 3.771; 95% CI 1.873–5.669; p = 0.027) and a low PNI (HR 2.351; 95% CI 1.026–3.676; p = 0.041) were found to be independently associated with poor survival.

**Table 1:** Prognostic factors for cancer-specific survival in 245 gastric cancer patients after gastrectomy.

Variable	Patients	Characteristics	Univariate			Multivariate		
			HR	95% CI	p value	HR	95% CI	p value
Gender	179/66	M/F	0.437	0.258–0.616	0.773			
Age (year)	162/83	< 65/≥ 65	2.116	0.493–3.739	0.042	1.217	0.435–1.999	0.076
Differentiation	161/84	Well and moderate/poor	3.542	0.764–6.320	0.037	2.784	1.218–4.350	0.218
TNM stage	99/146	I + II + III + IV	4.378	2.581–6.175	0.025	3.771	1.873–5.669	0.027
Vessel invasion	126/119	N/P	1.339	0.337–2.341	0.189			
Lymph node metastasis	73/172	N/P	2.392	0.469–4.315	0.028	0.685	0.437–0.933	0.133
PNI	148/97	< 43/≥ 43	2.573	0.692–4.454	0.033	2.351	1.026–3.676	0.041

### Relationships between PNI and clinicopathological features in non-elderly patients

**Figure 3:**
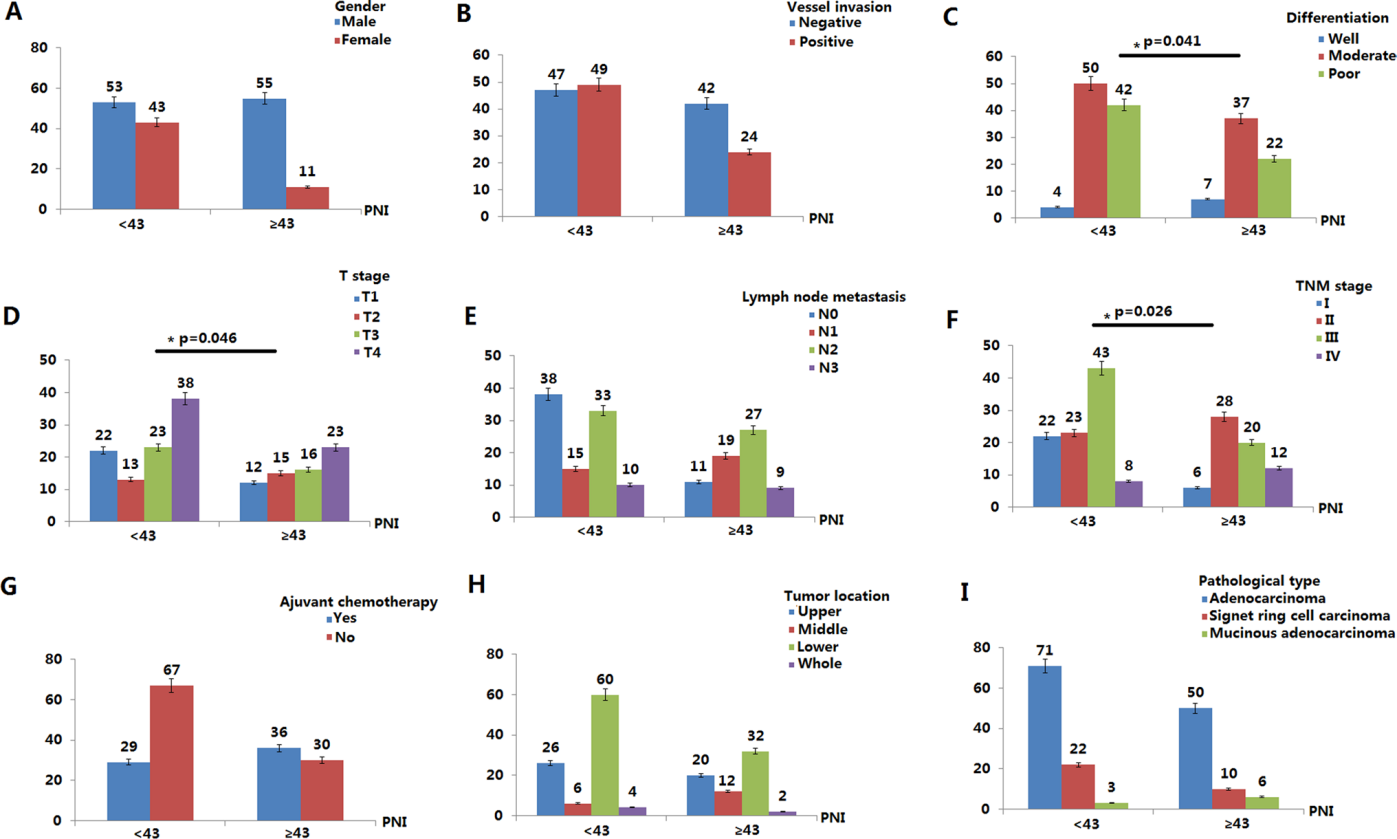
Relationship between PNI and clinicopathological features in 162 non-elderly (< 65) gastric cancer patients after total gastrectomy. There was a significant correlation between PNI and lymphocyte infiltration (p = 0.034), cancer differentiation (p = 0.041), TNM stage (p = 0.026) and T stage (p = 0.046) in non-elderly patients (< 65).

There was a significant correlation between PNI and lymphocyte infiltration (p = 0.034), cancer differentiation (p = 0.041), TNM stage (p = 0.026) and T stage (p = 0.046) in non-elderly patients (< 65) (Fig. 3). Univariate analysis showed that TNM stage (HR 3.223; 95% CI 1.002–5.444; p = 0.019), vascular invasion (HR 1.982; 95% CI 0.649–3.315; p = 0.041), lymph node metastasis (HR 1.794; 95% CI 0.364–3.224; p = 0.044) and low PNI (HR 2.018; 95% CI 0.357–3.679; p = 0.048) were important risk factors for poor prognosis (Table 2). In multivariate analysis, TNM stage (HR 3.116; 95% CI 1.235–4.997; p = 0.027) and low PNI (HR 2.034; 95% CI 0.337–3.731; p = 0.037) were independently related to poor survival time (Table 2). In a comparative study of PNI value and survival analysis in non-elderly patients after total gastrectomy, low PNI and short disease-free survival were statistically associated (p = 0.022), but low PNI and short overall survival time was not statistically correlated (Table 3).

**Table 2:** Prognostic factors for cancer-specific survival in 162 non-elderly (< 65) gastric patients after total gastrectomy.

Variable	Patients	Characteristics	Univariate			Multivariate		
			HR	95% CI	p value	HR	95% CI	p value
Gender	108/54	M/F	0.665	0.226–1.104	0.553			
Differentiation	98/64	Well + moderate/poor	2.376	0.218–1.746	0.211			
TNM stage	79/83	I + II/III + IV	3.223	1.002–5.444	0.019	3.116	1.235–4.997	0.027
Vessel invasion	109/53	N/P	1.982	0.649–3.315	0.041	1.229	0.562–1.896	0.195
Lymph node metastasis	89/73	N/P	1.794	0.364–3.224	0.044	0.783	0.337–1.229	0.169
PNI	96/66	< 43/≥ 43	2.018	0.357–3.679	0.048	2.034	0.337–3.731	0.037

**Table 3:** Prognostic role of PNI on 162 non-elderly (< 65) gastric cancer after total gastrectomy.

Characteristics	Patients	DFS (M)	p value	χ^2^	OS (M)	p value	χ^2^
PNI			0.022	4.47		0.278	2.745
< 43	96	28.7			37.2		
≥ 43	66	31.7			38.18		

### Relationships between PNI and clinicopathological features in elderly patients

**Figure 4:**
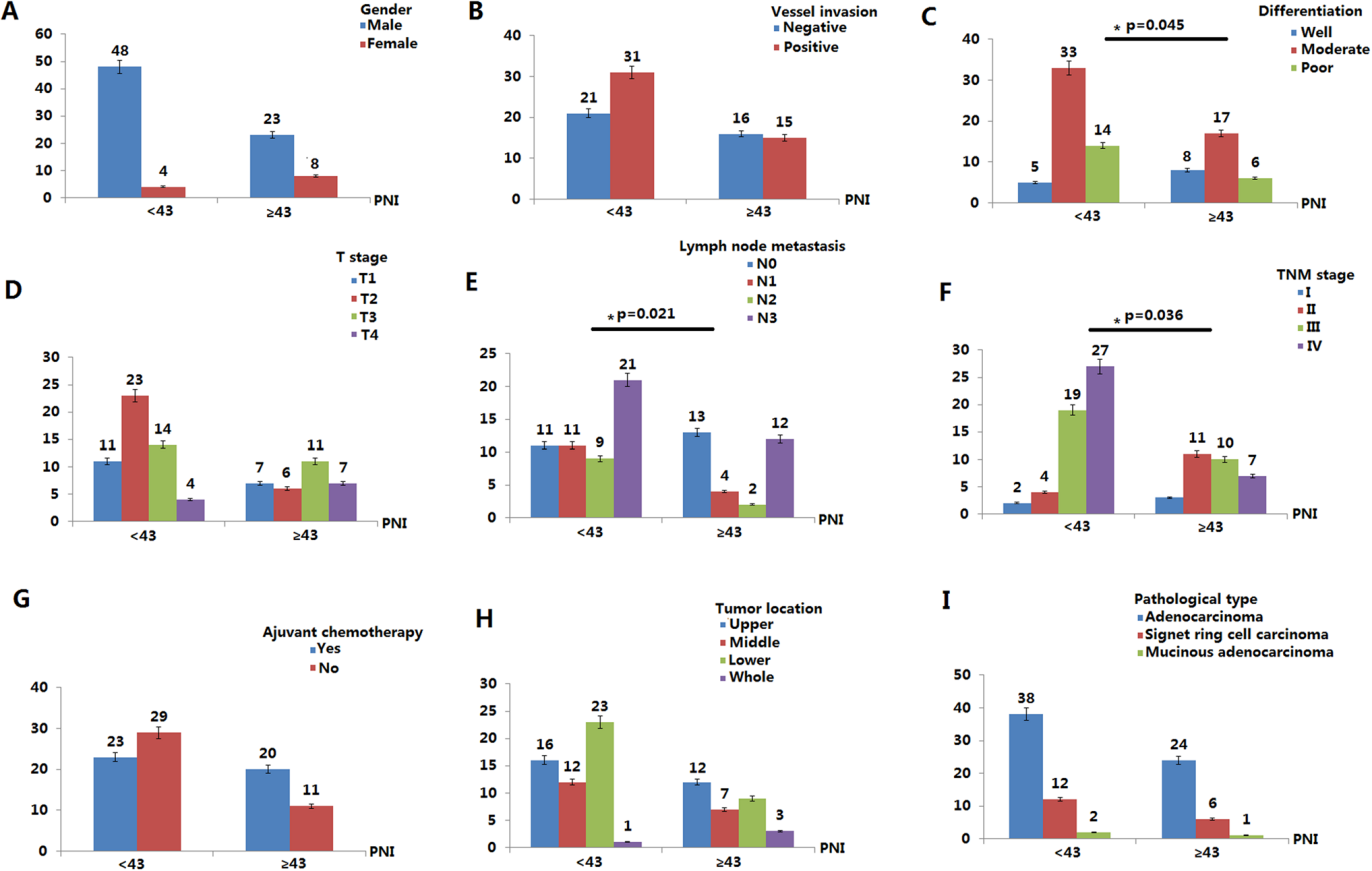
Relationship between PNI and clinicopathological features in 83 elderly (≥ 65) gastric cancer patients after total gastrectomy. In elderly patients (≥ 65), there was a significant correlation between PNI and lymphocyte infiltration (p = 0.021), cancer differentiation (p = 0.045) and TNM stage (p = 0.036).

In elderly patients (≥ 65), there was a significant correlation between PNI and lymphocyte infiltration (p = 0.021), cancer differentiation (p = 0.045) and TNM stage (p = 0.036) (Fig. 4). Univariate analysis showed that TNM stage (HR 3.381; 95% CI 1.275–5.487; p = 0.036), tumor differentiation (HR 2.256; 95% CI 0.542–3.970; p = 0.033) , lymph node metastasis (HR 2.218; 95% CI 0.562–3.874; p = 0.041) and low PNI (HR 2.229; 95% CI 0.783–3.675; p = 0.027) were important risk factors for poor prognosis (Table 4); In multivariate analysis, TNM stage (HR 2.968; 95% CI 0.723–5.213; p = 0.032) and low PNI (HR 2.427 95% CI 0.573–4.281; p = 0.028) were independently associated with poor survival time (Table 4). In a comparative study of PNI value and survival analysis in elderly patients after total gastrectomy, there were statistical correlation between low PNI and short disease-free survival time (p = 0.036) and short overall survival time as well (p = 0.047) (Table 5).

**Table 4:** Prognostic factors for cancer-specific survival in 83 elderly (≥ 65) gastric cancer patients after total gastrectomy.

Variable	Patients	Characteristics	Univariate			Multivariate		
			HR	95% CI	p value	HR	95% CI	p value
Gender	71 vs 12	M/F	0.682	0.117–1.247	0.773			
Differentiation	63/20	Well + moderate/poor	2.256	0.542–3.970	0.033	1.562	0.554–2.570	0.167
TNM stage	26/63	I + II/III + IV	3.381	1.275–5.487	0.036	2.968	0.723–5.213	0.032
Vessel invasion	37/46	N/P	1.265	0.115–2.415	0.189			
Lymph node metastasis	38/45	N/P	2.218	0.562–3.874	0.041	0.829	0.331–1.327	0.329
PNI	52/31	< 43/≥ 43	2.229	0.783–3.675	0.027	2.427	0.573–4.281	0.028

**Table 5:** Prognostic role of PNI on 83 elderly (≥ 65) gastric cancer after total gastrectomy.

Characteristics	Patients	DFS (M)	p value	χ^2^	OS (M)	p value	χ^2^
PNI			0.036	3.672		0.047	4.892
< 43	52	25.7			30.7		
≥ 43	31	30.6			34.2		

### Statistical analysis of PNI on survival parameters

**Figure 5:**
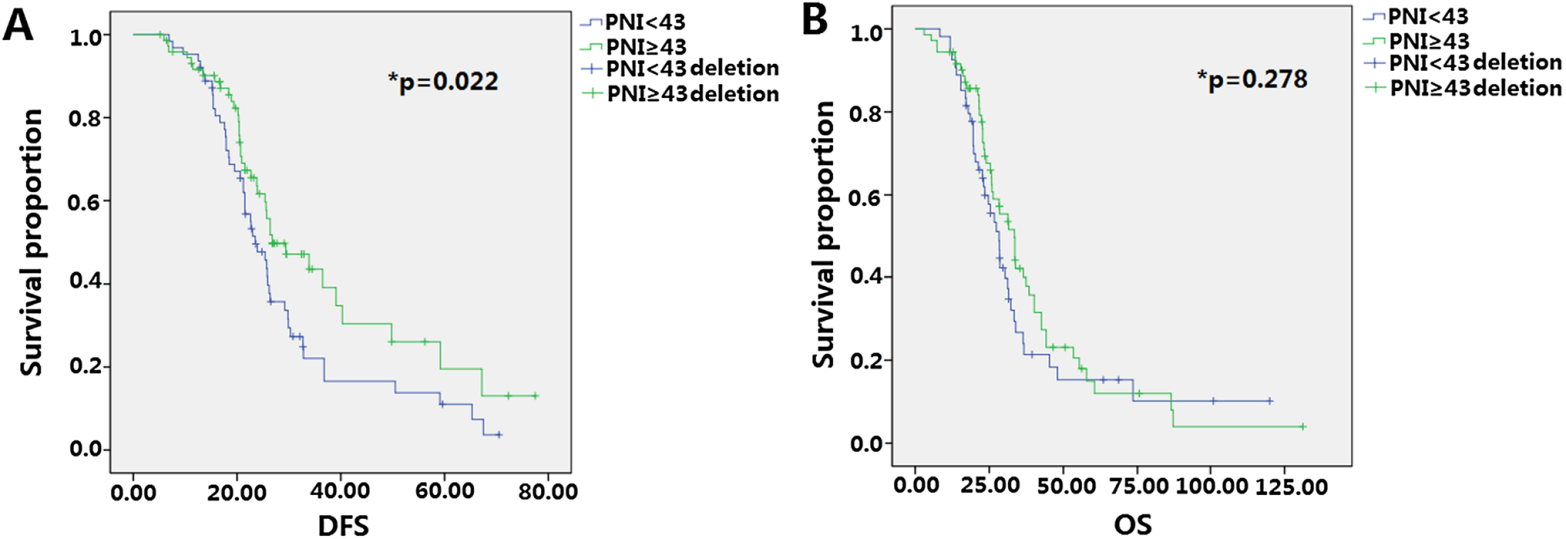
Predictive analysis of PNI on DFS and OS after total gastrectomy on 162 non-elderly (< 65) patients with gastric cancer. (**A**) The effect of PNI level on DFS of non-elderly patients (< 65), low PNI value is associated with shorter DFS and it has statistical significance; (**B**) the effect of PNI level on OS of non-elderly patients (< 65), low PNI value is associated with shorter OS and it has no statistical significance.

**Figure 6:**
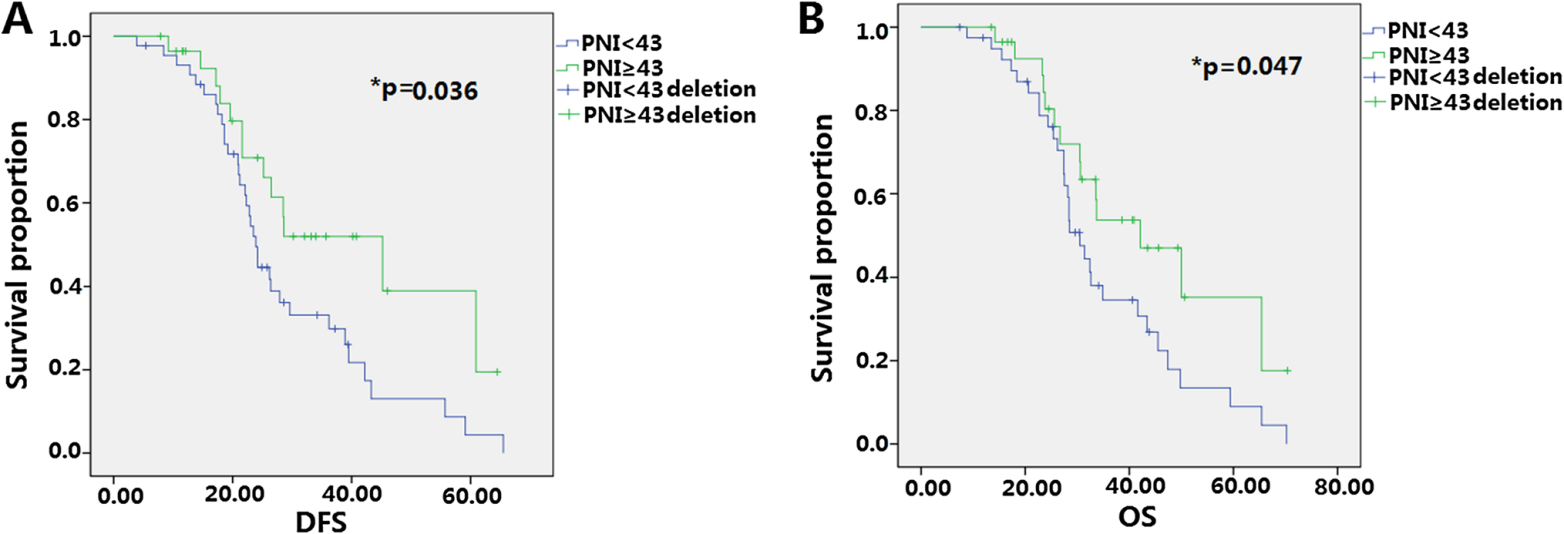
Predictive analysis of PNI on DFS and OS after total gastrectomy on 83 elderly (≥ 65) patients with gastric cancer. (**A**) The effect of PNI level on DFS of elderly patients (≥ 65), low PNI value is associated with short DFS and it has statistical significance; (**B**) the effect of PNI level on OS of elderly patients (≥ 65), low PNI value is associated with short OS and it has statistical significance.

We then analyzed the pre-operative PNI values of 245 patients and divided them into PNI < 43 and PNI ≥ 43 groups. As shown in Fig. 5, in non-elderly patients (< 65), low PNI is an independent prognostic factor for a short DFS; in elderly patients (≥ 65), low PNI is an independent prognostic factor for a short DFS and OS (Fig. 6).

## Discussion

The tumor microenvironment plays an important role in the process of tumorigenesis. Immune and nutritional status, as parts of tumor microcirculation, will undoubtedly affect the prognosis of patients. More and more evidence show that basic nutritional status and systemic inflammation are related to the long-term prognosis of cancer patients^16–22^. Malnutrition and low immune function not only affect the treatment effect of patients with malignant tumors, but also make malignant tumors more prone to relapse and metastasis^17^. More and more researches show that the nutritional status and immune function in patients with malignant tumors are closely linked to prognosis^18^. Compared with patients with normal nutritional status and immune function, the prognosis of those with poor nutritional status and immune function are also poor. A large number of studies have shown that immune nutrition status can be used as a powerful indicator to predict the survival outcome of patients with malignant tumors^19–22^.

Onodera first confirmed the prognostic role of PNI in gastrointestinal surgery of malnourished cancer patients in 1984^23^. Recently, many studies have shown that pre-operative PNI is a good predictor of cancer prognosis after cancer surgery^7,8,17,24–26^. We in this study investigated the relatively homogeneous group of stage I–IV gastric cancer patients undergone total gastrectomy to avoid non-uniformities which undermine the scientific interpretation of PNI in reality as shown by most of the published researches.

We found that compared with patients with PNI ≥ 43, there was a significant relationship between PNI < 43 and poor median OS. After analysis and calculation, the cut-off value of pre-operative PNI was defined to be 43, which was close to the average value after the normal test. It needs to be pointed out that though there are a lot of articles about PNI published, the cut-off value of it is different in each research. This is understandable, because PNI is a dynamically changing individual indicator in the cancer microenvironment. Therefore, research of PNI in a specific cancer type, among a specific population, and under a specific state is more meaningful. Yurday et al. reported that PNI was a robust novel prognostic factor that stratifies patients with stage IIIB NSCLC and they found the cut-off value of 40.5 was statistically meaningful in the prognosis of patients survival time^27^. In a study about PNI prognosis in patients with high-grade serous ovarian cancer (HGSC), Zheng et al*.* found that a low preoperative PNI (< 45.45) was associated with an advanced FIGO stage, increased CA125 level, more extensive ascites, residual disease and platinum resistance^28^. In general, the cutoff value of PNI fluctuated between 45 to 57 according to the already published researches^29–33^. Refining the cutoff value for different cancer type and individual patients is an area of active research.

Although it is unclear how PNI affects the exact mechanism of cancer outcomes, the prognostic value of PNI in cancer full management is certain^34–36^. It is hypothesized that patients with high PNI may have the appropriate general conditions, as result, they can be easily presumed to have better compliance at treatment, which could make difference in long term oncologic outcomes. Gastric cancer is a malignant tumor related to digestion and nutrition. It is more significant to discuss the role of nutritional factors in the process and treatment of it. We here in this study showed TNM stage, patients age, lymph node metastasis, tumor differentiation and a low PNI were significant risk factors for a poor prognosis by univariate analyses and TNM stage, patients age and a low PNI were found to be independently associated with poor survival in multivariate analysis. When we divide the patients into non-elderly and elderly groups, significant associations were found between the PNI and factors such as lymphocyte invasion, cancer differentiation, TNM stage and tumor infiltration in non-elderly patients and the lower PNI was correlated with shorter DFS in non-elderly patients; while in elderly patients, lymphocyte invasion, cancer differentiation and TNM stage were also statistically significant and the lower PNI was correlated with shorter DFS and OS. From another point of view, nutritional parameters have been reported to be related to sensitivity to treatment^37^, so, the above results can help us to choose the potential beneficiary from the adjuvant treatment after total gastrectomy which is significant in clinical management.

This study included some patients with stage IV gastric cancer, and there has been no widely accepted operational indication of total gastrectomy for such patients. In a sense, the clinical characteristics and prognosis of stage IV patients are crucial to the results of this study. In general, the following patients are suitable for total gastrectomy according to our research: Firstly, the patients only have positive peritoneal cytology who are relatively mild in stage IV group, and their prognosis is significantly better than those with extensive metastases; Secondly, the patients only have proximal organ metastasis who are cautiously discussed through multidisciplinary consultation; Thirdly, the patients with metastases under special circumstances after weighing the pros and cons, for example, some patients with partial local infiltration into the pancreas body may have indications for surgery, but patients with infiltration into the pancreatic head generally lose the opportunity for surgery.

In addition to PNI, the other two commonly used nutritional parameters in the prognosis of gastric cancer are the nutritional risk index and geriatric nutritional risk index (GNRI). Both of them can be used to judge whether the patient has a good general physical condition or not. It is generally believed that patients with good nutritional status have better tolerance to the treatment and thus have a better clinical prognosis. Nutritional factors play an important role in gastric cancer prognosis and nutritional status are more likely to be a reflection of the imbalance of the tumor microenvironment in vivo*.*

The pre-operative PNI also has prognostic value for other types of gastrectomy. Jee et al.^38^ retrospectively reviewed a prospectively maintained database of 7781 gastric cancer patients who underwent gastrectomy from January 2001 to December 2010 at a single center. From that data, they analyzed clinical and pathological characteristics, PNI, and short- and long-term surgical outcomes for each patient. They found that low PNI was a poor prognostic factor for overall survival with any kind of gastrectomy and PNI can be used to predict patients at increased risk of post-operative morbidity and mortality.

Some limitations of the present study need to be noticed, Firstly, this is a retrospective study and the limited single study institute and population need to be enlarged in the future study; Secondly, patients with neo-adjuvant therapy were excluded in this study to ensure that all patients are in the same state before blood sampling, this excludes the difference between the West where neo-adjuvant treatment is routine practice and the East where neo-adjuvant treatment is not so common^39^. Therefore, the results of this study are not applicable to gastric patients undergoing neo-adjuvant therapy; Finally, the last one but certainly not the least one, PNI is a dynamic indicator, the previous controversy on its critical point is largely due to the difference in the nutritional status of patients in different disease states and treatment states, so it is necessary to distinguish between the early, advanced and inoperable state or different stratifications such as part resection, total resection and resection together with adjacent organs removal among the gastric cancer patients which may be more clinically valuable.

The pre-operative PNI can better reflect the surgical risk and nutritional status of gastric cancer patients. Low PNI is an independent risk factor for poor prognosis in gastric cancer patients (Supplementary Fig. 2). Therefore, patients with low pre-operative PNI levels should be observed more closely after surgery to avoid the occurrence of post-operative complications in the near future. At the same time, more detailed and closed long-term follow-up should be placed on these patients in order to obtain the opportunity to intervene in the relapse or metastasis as early as possible.

## Methods

### Patients

A retrospective analysis was conducted of 245 gastric cancer patients underwent total gastrectomy with R0 resection in Peking University, First Hospital between January 1st 2005 and December 30th 2015. R0 resection is defined as complete resection with negative margin. The inclusion criteria were included: (1) gastric cancer confirmed by histology and pathology; (2) clinical stage confirmed according to the 8th edition; (3) ECOG (Eastern Cooperative Oncology Group) performance status score of 0–1; (4) proportion of body/mass ≥ 20.0 kg/m^2^; (5) without history of other cancer; (6) no neo-adjuvant radiotherapy or chemotherapy; (7) available blood tests results collected before surgery. The exclusion criteria were included: (1) receiving any kinds of therapies before the operation; (2) pre-operative death; (3) loss of follow-up; (4) no pre-operative blood cell counts records; (5) concurrent infection; (6) autoimmune disease.

We collected the clinicopathological data and laboratory records from the patient’s case history. The patients were followed up in Peking University, First Hospital and end points for the investigation were disease-free survival (DFS) and overall survival (OS). OS was defined as the length of time from randomization to death for any reasons after total gastrectomy. DFS was defined as the time between the beginning of randomization to the recurrence of the disease or death for any causes. The end point follow-up was placed on March 2020.

Patients gave their written informed consent. The study protocol was approved by the institutional committee on human research of the Institutional Review Board (IRB) of Peking University, First Hospital. We confirm that all methods were performed in accordance with the relevant guidelines and regulations proved by IRB of Peking University, First Hospital.

### Prognostic Nutritional Index (PNI)

PNI = (10 × serum Albumin, g/dL) + (0.005 × blood lymphocyte count, unit/L). Blood samples were obtained at a maximum period of 2 weeks before gastrectomy due to the half lives of albumin (≈ 21 days) and lymphocytes (> 2 weeks). The cut-off value of PNI was measured by the maximum Youden index (sensitivity + specificity − 1) in the time dependent receiver operating characteristics (ROC) curve for recurrence and survival according to published literature^14^.

### Statistical analyses

Statistical analysis was performed using SPSS 20.0 software, chi-square test was used for comparison of probability calculation. The spearman test was used for correlation analysis. Survival rate was calculated by Kaplan–Meier survival curve, log-rank test was used for univariate analysis, and COX regression was used for multivariate analysis. p < 0.05 meant the difference was statistically significant.

## Supplementary information

Supplementary Figure 1.

Supplementary Figure 2.

Supplementary Information.
