# Supplementary figures and images for: The role of prognostic nutritional index for clinical outcomes of gastric cancer after total gastrectomy
Source: Sci Rep. 2020 Oct 15;10:17373. doi: 10.1038/s41598-020-74525-8 (PMC7562903; doi:10.1038/s41598-020-74525-8)

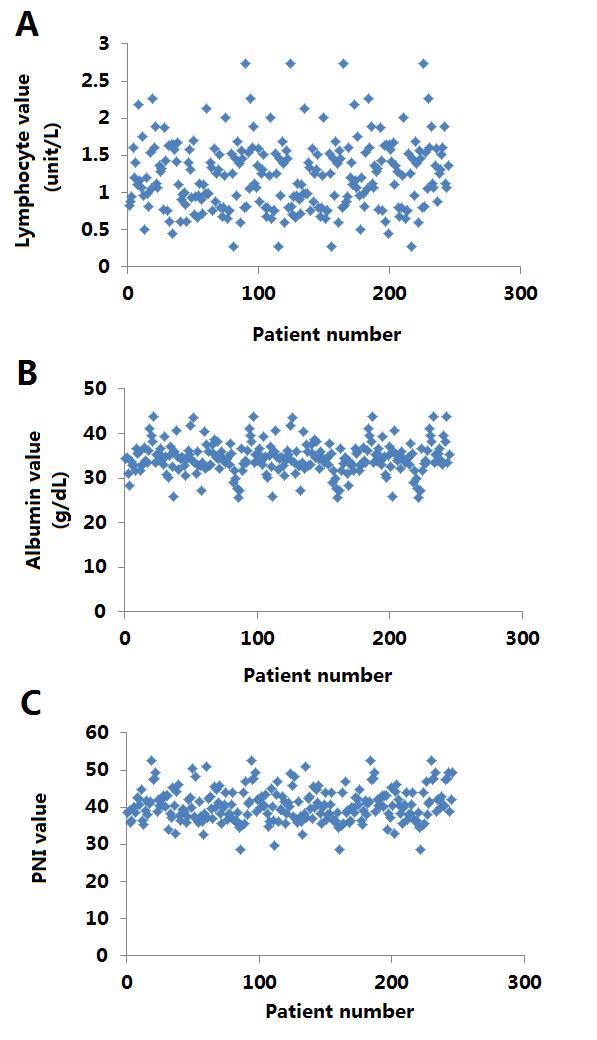

Supplement: Supplementary file 1 — Supplementary Figure 1. [file 41598_2020_74525_MOESM1_ESM.png]

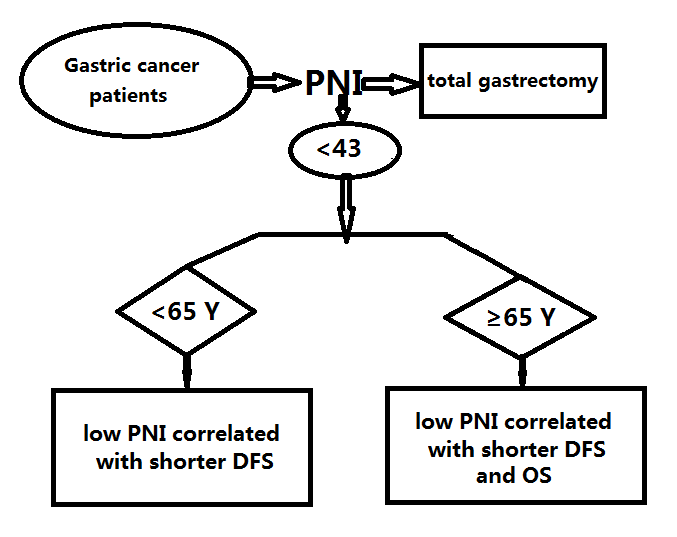

Supplement: Supplementary file 2 — Supplementary Figure 2. [file 41598_2020_74525_MOESM2_ESM.png]
