# Supplementary material for: The role of prognostic nutritional index for clinical outcomes of gastric cancer after total gastrectomy
Source: Sci Rep. 2020 Oct 15;10:17373. doi: 10.1038/s41598-020-74525-8 (PMC7562903; doi:10.1038/s41598-020-74525-8)
Supplement: Supplementary file 3 — Supplementary Information. [file 41598_2020_74525_MOESM3_ESM.docx]

**Supplementary Figure1 The flow data of the hematological index. A.**The lymphocyte value (unit/L) ranged from 0.27 to 2.74, with a median level of 1.54. **B.** The albumin value (g/dL) ranged from 25.7 to 43.8, with a median level of 34.3.**C.** The PNI ranged from 33.9 to 52.4, with a median level of 45.7 and the optimal cut-off point of the PNI was 43.15.

**Supplementary Figure2 The schematic representation of the whole outcome of this study.** The pre-operative PNI can better reflect the surgical risk and nutritional status of gastric cancer patients. Low PNI is an independent risk factor for poor prognosis in gastric cancer patients. Low PNI correlated with shorter DFS in non-elderly(<65) patients and shorter DFS and OS in elderly(≥65) patients.
